# 

**Published:** 1887-06-18

**Authors:** 


					Contents.
A Treadling Matcli: The Sunday Fund
193
Words of Consolation.-
-XXXVII.
The Work of the
Holy uhost
Counterparts.
tiv a bTUDENT OF ^ASTERN rHILO-
Chap. II
Facts about Hospitals;
for Preachers, etc.
By
Henry C. Burdett
197
Tlie Metropolitan Hospital Sunday Fund.
By Sir Sydney Waterlow, Bt., Vice-President of the
Council -------
Notes and Sews.-
-Prince Albert Victor and the
Donnybrook Hospital.?Entertainments in Aid of the
Hospitals.?Hospital Enlargement.?Vacancies, etc., etc.
Annotations.
-Who cares for the Pauper Children ??
A New Lunatic Asylum for Middlesex. ? Good
Women -------
203
A Crusade against Indifference -
Poetry.-
-Hospital Sunday -
Traill e?l Nurses' Fund -
206
Tlie Cliildi't'ii'K Corner.?Puzzles, Answers, etc.
Amusements wltli Profit
Iii- and Out-Patients' Hospital Diary - 207

				

